# The successful combination of grapefruit juice and venetoclax in an unfit acute myeloid leukemia patient with adverse risk: A case report

**DOI:** 10.3389/fonc.2022.912696

**Published:** 2022-09-28

**Authors:** Zhangbiao Long, Min Ruan, Wei Wu, Qingshu Zeng, Qingsheng Li, Zhengqi Huang

**Affiliations:** Department of Hematology, The First Affiliated Hospital of Anhui Medical University, Hefei, China

**Keywords:** grapefruit, venetoclax, AML – acute myeloid leukaemia, unfit, adverse risk

## Abstract

Venetoclax combined with hypomethylating agents such as azacitidine and decitabine is the standard regime for the elderly patient with acute myeloid leukemia (AML) unfit for intensive induction therapy. However, many patients struggle with finances and forgo treatments due to the high costs of venetoclax. In this study, we performed the regime with azacitidine, low-dose venetoclax, and grapefruit juice on an unfit AML patient with TP53 mutation. The peak venetoclax concentration (C_max_) and side effects on the patient were also monitored. The patient achieved complete remission with the venetoclax C_max_ within the effective concentration range (1,000–3,000 ng/ml) and maintained durable remission until recently. Febrile neutropenia, thrombocytopenia, and pneumonia appeared during the first cycle and were recovered by stimulating agents and antibiotic treatment. This improvement combination approach by drug-food interaction may enlighten other similarly patients with AML, especially those in low-middle income countries.

## Introduction

Elderly patients with acute myeloid leukemia (AML) have a dismal outcome and cannot tolerate conventional intensive induction chemotherapy. Taking the place of supportive care, venetoclax combination with hypomethylating agents such as azacitidine and decitabine is currently becoming the preferred regime for these patients (1). Previous studies investigated the overall response rate was approximately 66–73%, and the median overall survival was 14.7–17.5 months in *de novo* unfit patients with AML (2–4). In recent years, venetoclax has been approved in China and other countries for elderly patients with AML unfit for intensive induction therapy. Actually, the price remains too expensive (5). For instance, the cost of venetoclax is currently 38,880 RMB yuan per month in China. Due to the high costs of venetoclax, many patients face considerable financial struggles. These patients usually forgo treatments and succumb to death at last.

A strategy to reduce the cost of venetoclax is dose reduction. Venetoclax is an oral agent, primarily metabolized by cytochrome P450 enzyme (CYP3A4) (6). Several previous studies revealed that concurrent use of CYP3A4 inhibitors such as azole antifungal agents and venetoclax could reduce the metabolism of venetoclax, thus increasing its exposure (7, 8). The recommended dose of venetoclax is 100 mg once daily for patients receiving a strong concomitant CYP3A4 inhibitor (i.e., posaconazole or voriconazole) (9). When the venetoclax dose was reduced to 100 mg once daily from 400 mg once daily, the cost of venetoclax reduced to 9720 RMB yuan per month. Although some researchers recommended antifungal treatment for AML patients with grade 4 neutropenia, only a portion of patients with AML required azole antifungal treatment or prophylaxis during the first few cycles (3, 10–12).

Some foods can be considered a practical approach to enhancing the plasma concentration and effect of CYP3A4 substrates. Grapefruit juice is the most common food that can strongly inhibit CYP3A4 (13, 14). The simultaneous use of grapefruit juice and CYP3A4 substrate was an advanced strategy and also a challenge in clinical management. Here, we present an unfit newly diagnosed patient with AML who has low income and could not tolerate the high price of venetoclax. She has adopted the solution with the combination of grapefruit juice 200 ml 3 times daily, venetoclax 100 mg once daily, and azacitidine 75 mg/m^2^ on days 1–7 of each 28-day cycle. We detected the patient’s peak venetoclax concentration (C_max_) to ensure appropriate drug exposure and reduce drug toxicity. In addition, we monitored the QTc interval, liver function, and renal function to avoid severe side effect. Eventually, the patient achieved complete remission and sustained durable remission until this present.

## Case report

**Figure 1:**
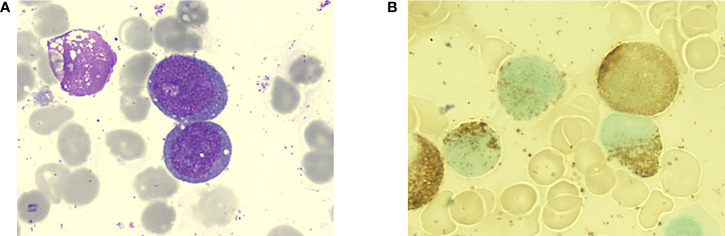
Bone marrow aspiration of the patient at the initial diagnosis. **(A)** Large-sized myeloid blasts with slight nuclear irregularities and scant granular cytoplasm (Wright-Giemsa staining, 1000×). **(B)** Peroxidase staining of the blasts was positive.

The patient was a 70-year-old woman. In July 2021, she came to the outpatient department of our hospital because of progressive dizziness and fatigue for 3 months without any cause. Initial blood routine test results showed pancytopenia (**Table 1**). A bone marrow aspiration was performed at our hospital. The bone marrow smears showed 42.5% large-sized blasts with fine chromatin and occasional prominent nucleoli (**Figure 1**). Flow cytometry of the bone marrow revealed positive for CD34, HLA-DR, myeloperoxidase, CD33, CD13, CD117, and CD7 and negative for Cy79α, CD19, CyCD3, and CD5. Cytogenetic analysis of the bone marrow identified a normal karyotype (46, XX) without a (15;17) chromosomal translocation. Next-generation sequencing of the bone marrow showed a frameshift mutation in TP53 (NM_000546: exon5: c.388dupC: p.Leu130fs) and did not identify a mutation in other genes. The MICM results of bone marrow were consistent with the WHO classification of AML, not otherwise specified.

**Table 1:** The baseline laboratory data of the patient.

Variable	Baseline value	Reference range
White blood cell count (/ml)	810	3500-9500
Differential count (%)		
Neutrophils	23.5	40-75
Lymphocytes	64.2	20-50
Eosinophils	0	0.4-8
Basophils	1.2	0-1
Monocytes	11.1	3-10
Hemoglobin (g/dl)	8.2	11.5-15
Platelet count (/ml)	77000	125000-350000
Alanine aminotransferase (U/L)	16	7-40
Aspartate aminotranferase (U/L)	15	13-35
Alkaline aminotransferase (U/L)	62	35-100
Albumin (g/L)	40.4	40-55
Globulin (g/L)	21.1	20-40
Lactate dehydrogenase (U/L)	212	120-250
Uric acid (μmol/L)	257	155-357
Urea (mmol/L)	5.08	2.6-7.5
Creatinine (μmol/L)	48	41-73

The patient was generally evaluated for the subsequent treatment. The AML risk stratification by the genetics of this patient was adverse due to TP53 mutation. The Eastern Cooperative Oncology Group (ECOG) performance status was 2. The age and current situation suggested that the patient was ineligible for intensive chemotherapy. The preferred therapy of the patient was a hypomethylating agent combined with venetolcax. However, the patient was poor and could not afford the regular venetoclax dose of 400 mg once daily. After the patient’s agreement, informed consent was obtained from the patient and the approval of the Ethics committee of our hospital. We performed the improved therapy with the combination of azacitidine (75 mg/m^2^ subcutaneously on days 1–7 of each 28-day cycle), venetoclax (100 mg orally once daily), and grapefruit juice (200 ml orally 3 times daily) for this patient. To ensure the effectiveness of the reduced dose of venetoclax, we monitored the patient’s peak venetoclax concentration (C_max_) every week in the first cycle. The venetoclax C_max_ was 1,440 ng/ml at 7 days and 1,920 ng/ml at 14 days after the treatment and was within the effective concentration range (1,000–3000 ng/ml) (8, 15). In consequence, adherence to this regime was processed in the treatment. Febrile neutropenia and thrombocytopenia with grade 4 appeared 2 weeks after the therapy and completely recovered rapidly after the treatment with stimulating agents. The patient got pneumonia on day 14 after the initiation of treatment and recovered after the antibiotic treatment. The laboratory tests, including blood cell counting, liver function, renal function, and ECG were performed during the entire treatment period to monitor the side effect. The liver and renal functions were normal, and the QTc interval was 450 ms.

**Figure 2:**
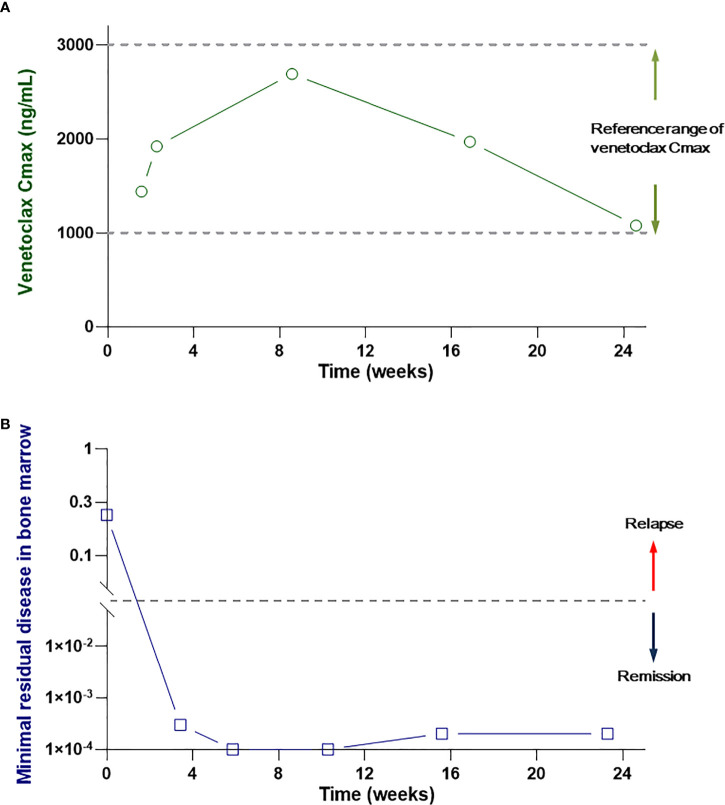
**(A)** The peak venetoclax concentration in the patient’s serum. **(B)** MRD in the patient’s bone marrow during the treatment and follow-up period.

The bone marrow aspiration was performed on day 21. The bone marrow smears and immunology results revealed normal without leukemia cells. The patient continued this regime for five cycles to the current presentation and sustained durable remission. There was always no morphologic or phenotypic evidence of AML relapsed (**Figure 2**). A notable side effect was not detected during the subsequent treatment and follow-up period.

## Discussion

Along with the application of novel target drugs, the management strategy of AML patients who were unfit for intensive induction therapy has changed from previous best supportive care to the regime of which combination of multiple targeted drugs. As a selective inhibitor of B cell lymphoma 2 regulatory protein (Bcl-2), venetoclax has become a backbone drug in the regime for unfit AML patients. It can be combined with hypomethylating agents or reduced dose chemotherapy, such as low-dose cytarabine, resulting in a high-response rate and prolonged overall survival in unfit AML patients (16). Venetoclax has been approved by the Chinese national medical products administration but has not been covered by Chinese medical insurance. Thus, reducing the costs that enable venetoclax to be applicable to many low-income patients is a challenge for clinical practice. In this present case, we added grapefruit juice to the regime venetolcax plus azacitidine and carefully monitored the concentration of venetoclax. The clinical data showed that grapefruit juice could effectively improve the C_max_ of venetoclax and obtain an ideal clinical effect. So it is a successful treatment for this patient.

Based on previous studies, the venetoclax concentration was positively correlated with the AUC curve and reflected the venetoclax exposure (7, 17). In this study, grapefruit juice can significantly increase the C_max_ of venetoclax and improve its effectiveness. We avoided the repeated detection of venetoclax concentration at multiple time points daily to reduce the iatrogenic anemia and cost. In the subsequent clinical trials, it can be considered to verify the pharmacokinetics of venetoclax by detecting the concentration at several time points. For this patient, we have significantly reduced the treatment cost. For other patients taking a sufficient dose of venetoclax, the current monthly cost is 38,880 RMB yuan, whereas that of this patient was 9,720 RMB yuan.

To monitor the side effects caused by grapefruit juice during the therapy period, we detected laboratory and imaging examinations, such as ECG. There was no QTc interval prolonged, and another abnormal result was found. The application of antibacterial was adequate for the infection of the patient. Thus, we did not use other CYP3A4 inhibitors in the whole treatment process. It is worth noting that if an azole antifungal agent such as posaconazole was used for prophylaxis or treatment, the application of grapefruit juice should be temporarily interrupted to avoid excessive venetoclax concentration. In addition, the concurrent use of azole antifungal agents also can decrease the venetoclax dose and reduce the costs, but not superior to grapefruit juice. There were several reasons. First, antifungal prophylaxis is not a universal recommendation but applies to patients with grade 4 neutropenia (neutrophil < 0.5 × 10^9^/L), whereas some AML patients without grade 4 neutropenia were not mandatory for antifungal prophylaxis (3, 10, 11, 18). Second, antifungal prophylaxis is usually applied during the myelosuppression period, ceased when recovery from neutropenia, rather than during the entire venetoclax plus azacitidine treatment period (10). Furthermore, a real-world observational study revealed significantly prolonged thrombocytopenia in AML patients with concomitant venetoclax and azole antifungals (9). Third, some institutions administer non-azole antifungal prophylaxis for AML patients rather than azole prophylaxis (19). In a retrospective study of 119 patients with AML, only 49 (41%) patients received azole-based antifungal prophylaxis (12). Fourth, the azole agents are more expensive than grapefruit juice. Thus, the treatment costs actually increase when combined with azole antifungals.

The patient herein achieved complete remission and sustained durable remission after the treatment of this regime, but another unknown was how long will this regime’s effects last? Venetoclax has shown a promising remission effect in patients with AML, but these patients’ progression-free survival has not yet been satisfied (20, 21). The patient in this study was an older woman with adverse prognostic gene mutation. Therefore, after remission, allogeneic hematopoietic stem cell transplantation is a curable approach for the patient. During the treatment and follow-up period, monitoring minimal residual disease by flow cytometry, digital PCR, or qRT-PCR was essential to alert the recurrence. Furthermore, the patients had a frameshift mutation in TP53. As a preclinical study demonstrated, applying sublethal venetoclax in AML may enhance the risk of disease progression. In contrast, sufficiently lethal treatment could maximize the outcomes of AML patients with TP53 mutation (22). A higher dose venetoclax strategy may be applied in future treatment but remains a challenge due to the deficiency of clinical evidence. A previous study demonstrated that TP53 deficiency impairs sensitivity to combined venetoclax. High-dose idarubicin and co-targeting MCL1 could enhance venetoclax activity in AML (23). Therefore, a regime containing these agents can be an intelligent choice. Further integration of the TP53 target agent into the current administration may be an objective for future studies (24, 25).

There were several limits to this research. First, the patients achieved complete remission and kept relapse free during the follow-up period. However, the follow-up is not long enough to assess the long-term efficacy of this approach. Second, we did not detect the venetoclax concentration at multiple time points and thus, did not collect adequate parameters to evaluate the venetoclax pharmacokinetics. Third, a single case may not enough to demonstrate the clinical safety and efficacy of grapefruit juice plus dose-adjusted venetoclax, a large sample, and long-term clinical trial were needed to confirm the results.

To the best of our knowledge, this is the first case of the combination of CYP3A4 inhibitor food and venetoclax in an AML patient. The efficacy and side effects need to be verified in further studies. The costs and feasibility of the novel drugs should be considered by physicians, especially those in developing countries. Furthermore, the food–drug interaction model presented in this study may provide a reference for other disorders.

## Conclusions

In this study, we reported that a patient with AML who could not afford expensive costs achieved complete remission with acceptable side effect by the regime with azacitidine, low-dose venetoclax, and grapefruit juice. This combination approach may enlighten other similar patients, especially those in low-middle income countries.

## Data availability statement

The raw data supporting the conclusions of this article will be made available by the authors, without undue reservation.

## Ethics statement

This study was reviewed and approved by the ethics committee of the first affiliated hospital of Anhui medical university. The patients/participants provided their written informed consent to participate in this study. Written informed consent was obtained from the individual(s) for the publication of any potentially identifiable images or data included in this article.

## Author contributions

QL, ZH and ZL designed the study and wrote the manuscript. ZL, MR and QZ collected the data and treated the patient. MR and WW performed examination and helped analyze examinational data. All authors contributed to the article and approved the submitted version.

## Funding

This study was supported by grants from the National natural science foundation (81900118).

## Conflict of interest

The authors declare that the research was conducted in the absence of any commercial or financial relationships that could be construed as a potential conflict of interest.

## Publisher’s note

All claims expressed in this article are solely those of the authors and do not necessarily represent those of their affiliated organizations, or those of the publisher, the editors and the reviewers. Any product that may be evaluated in this article, or claim that may be made by its manufacturer, is not guaranteed or endorsed by the publisher.
